# Live-Attenuated Influenza Vaccine Effectiveness in Children From 2009 to 2015–2016: A Systematic Review and Meta-Analysis

**DOI:** 10.1093/ofid/ofx111

**Published:** 2017-07-24

**Authors:** Herve Caspard, Raburn M. Mallory, Jing Yu, Christopher S. Ambrose

**Affiliations:** MedImmune, Gaithersburg, Maryland

**Keywords:** children, influenza, live-attenuated influenza vaccine (LAIV), meta-analysis, vaccine effectiveness

## Abstract

**Background:**

This systematic review and meta-analysis describes and consolidates findings from all studies that assessed the effectiveness of live-attenuated influenza vaccine (LAIV) against laboratory-confirmed influenza since the 2009 pandemic in children and young adults.

**Methods:**

A MEDLINE search was conducted for articles published from January 1, 2010 to November 30, 2016. All original publications reporting an effectiveness estimate of LAIV against cases of influenza confirmed by reverse-transcription polymerase chain reaction or culture were retained for analysis. Effectiveness estimates were categorized by LAIV formulation (monovalent, trivalent, and quadrivalent) and strain (any influenza strain, A(H1N1)pdm09, A(H3N2), and B strains). Consolidated estimates were obtained with a random-effects model.

**Results:**

A total of 24 publications presenting 29 observational studies were retained for meta-analysis. Live-attenuated influenza vaccine was not shown to be effective against A(H1N1)pdm09 strains as a monovalent formulation in 2009–2010 or as a trivalent formulation from 2010–2011 to 2013–2014, but consolidated sample sizes were small. It was effective as a quadrivalent formulation but less effective than inactivated influenza vaccine (IIV). Live-attenuated influenza vaccine was consistently effective against B strains and matched A(H3N2) strains but was not shown to provide significant protection against mismatched A(H3N2) strains in 2014–2015.

**Conclusions:**

These findings confirm that effectiveness of LAIV against A(H1N1)pdm09 strains has been lower than IIV. A systematic investigation has been initiated to determine the root cause of the difference in effectiveness between pre- and postpandemic A(H1N1) vaccine strains and to identify a more consistently effective A(H1N1)pdm09 vaccine strain.

A live-attenuated influenza vaccine (LAIV) has been approved for use in pediatric populations of the United States, Canada, and Europe since 2003, 2011, and 2012, respectively. Regulatory approval of LAIV and the subsequent recommendations for use by national policy bodies were supported by 6 randomized, double-blind, placebo-controlled clinical studies [1–6]. These studies demonstrated significant protection with trivalent LAIV (LAIV3) against influenza A(H1N1), A(H3N2), and influenza B strains in children aged 6 months to 6 years. Three additional studies demonstrated greater efficacy of LAIV3 in children aged 6 months to 17 years versus inactivated influenza vaccine (IIV) [7–9]. A quadrivalent formulation of LAIV (LAIV4) replaced LAIV3 in the United States during the 2013–2014 influenza season and in most other countries in 2014–2015, with use of LAIV4 in all countries where licensed beginning in 2015–2016.

The influenza antigens contained in vaccine formulations are updated annually based on recommendations from the World Health Organization and national regulators. A major antigenic shift occurred in 2009 when the influenza A(H1N1) pandemic required the creation of a monovalent vaccine against the substantially different A/California/7/2009 strain. This strain was subsequently incorporated into seasonal trivalent and quadrivalent vaccine formulations starting in 2010.

In addition to the efficacy data generated by randomized controlled studies conducted before approval/licensure, observational studies—in which vaccine allocation was not controlled by the investigators—have evaluated vaccine effectiveness (VE) in multiple countries since the 2003–2004 season [10, 11]. Live-attenuated influenza vaccine-specific effectiveness against laboratory-confirmed influenza was first reported for the monovalent A(H1N1)pdm09 LAIV, which was distributed during the 2009 pandemic [12–14]. Estimates of LAIV effectiveness from 2009 to 2012–2013 were generally consistent with the results of randomized controlled studies, although the statistical power to detect differences versus IIV was limited. In June 2014, based on a review of the evidence on the relative efficacy and effectiveness of LAIV versus IIV, the US Centers for Disease Control and Prevention (CDC) Advisory Committee on Immunization Practices (ACIP) recommended that, when immediately available, LAIV4 should be used in healthy children aged 2–8 years who had no contraindications or precautions for use [15]. However, the ACIP did not renew this preferential recommendation for the 2015–2016 season, because a review of data from observational studies showed no significant effectiveness of LAIV4 against influenza A(H1N1)pdm09 in 2013–2014 [16]. In June 2016, the ACIP made the interim recommendation that LAIV4 should not be used in the 2016–2017 season after reviewing the findings of observational studies that showed reduced effectiveness of LAIV4 versus IIV in 2015–2016 [17]. The objective of this meta-analysis was to evaluate the effectiveness of LAIV in children against laboratory-confirmed influenza from 2009–2010 to 2015–2016.

## METHODS

### Search Strategy and Study Selection

A MEDLINE search was conducted for articles published from January 1, 2010 to November 30, 2016. Prespecified search terms included influenza, vaccine, and effectiveness. The terms 95% confidence interval (CI) and 95% CI were added secondarily. Terms could appear in either the article title or abstract. Additional information was identified from the websites of regulatory and public health agencies in countries in which LAIV is distributed, including presentations at the ACIP public meetings, the Public Health Agency of Canada, and the State Agency for Consumer Protection Saxony-Anhalt, Department of Hygiene, Magdeburg, Germany. When data from studies sponsored by MedImmune (the manufacturer of LAIV) were not yet published, data from publicly available reports submitted to the ACIP were reviewed.

References were then screened for data reporting effectiveness of a LAIV formulation that included an A(H1N1)pdm09 vaccine strain versus no vaccine against cases of influenza confirmed by cultures or reverse-transcription polymerase chain reaction (RT-PCR). Controlled clinical studies, reviews, meta-analyses, letters, and opinion pieces as well as references that did not mention LAIV in the full text were excluded. Eligible references were then assessed for inclusion in the meta-analysis. References were excluded if they (1) contained duplicate data from other publications, (2) studied a population that did not use LAIV, or (3) did not provide a LAIV-specific effectiveness estimate. The quality of evidence was assessed by a review of the study eligibility criteria, age range, and methods for VE adjustment, which are documented in Table 1.

**Table 1.** Characteristics of the Studies Retained for Meta-Analysis

Sponsor/Institution	Influenza Season	LAIV Formulation	Setting	Study Design	Eligibility Criteria	Age Range (Years)	Adjusted VE Estimate
Centers for Disease Control and Prevention (CDC)	2009–2010 [11]	A(H1N1)	Several sites across the USA (MI, PA, TN, TX, WA, WI)	TNCC	ARI	2–9	Yes
	2010–2011 [32]	Trivalent				2–8	
	2011–2012 [33]						
	2012–2013 [34]					2–17	
	2013–2014 [35]	Quadrivalent					
	2014–2015 [36]						
	2015–2016 [37]						
Maine Center for Disease Control and Prevention (Uzicanin)	2009–2010 [14]	A(H1N1) monovalent	Schools/Maine	Case control	Controls were healthy classmates	4–14	Yes
New York City Department of Health and Mental Hygiene (Hadler)	2009–2010 [13]	A(H1N1) monovalent	Hospitalized children/New York City	Case control	Controls from Immunization Registry	2–9	Matching on age and ZIP code
US Department of Defense	2010–2011 [38]	Trivalent	Active duty service members	TNCC	ARI	18–49 48% <25	Yes
	2011–2012 [39]		Active duty US service members			18–49 62% ≤28	
	2012–2013 [40]		Service members			2–49	
			Civilians/ dependents			2–49	
			Service members/ dependents			2–49	
	2013–2014 [41]	Quadrivalent	Service members/ dependents			2–49 43% <18	
	2015–2016 [37]	Quadrivalent	Service members/ dependents			2–17	
State Department of Hygiene, Magdeburg, Germany (Helmeke)	2012–2013 [42]	Trivalent	Primary care practices/ Germany	TNCC	ARI	2–17	Yes
	2015–2016 [43]	Quadrivalent					
Canada’s Sentinel Physician Surveillance Network (SPSN)	2013–2014 [44]	Trivalent	Community-based practitioners	TNCC	ARI	2–19	No
	2015–2016 [45]	Quadrivalent				2–17	No
University of Michigan School of Public Health, Ann Arbor (Ohmit)	2013–2014 [46]	Quadrivalent	232 households in Michigan	Cohort	—	2–17	Yes
MedImmune, USA	2013–2014 [47]	Quadrivalent	Several sites in the USA (FL, MN, NC, OH, OR, TN, TX, WI)	TNCC	FARI	2–17	Yes
	2014–2015 [48]						
	2015–2016 [49]						
Public Health England (PHE)	2014–2015 [50]	Quadrivalent	Primary care practices	TNCC	ILI	2–7	Yes
	2015–2016 [51]						
National Institute for Health and Welfare, Finland (Nohynek)	2015–2016 [52]	Quadrivalent	Nationwide register	Cohort	—	Birth cohort of 2013	Yes

Abbreviations: ARI, acute respiratory infection; FARI, febrile acute respiratory infection; ILI, influenza-like illness; LAIV, live-attenuated influenza vaccine; TNCC, test-negative case control; VE, vaccine effectiveness.

Studies were assessed for inclusion by 2 authors (H.C. and J.Y.), and ambiguous decisions were resolved by consultation with another author (C.S.A.). The primary authors of the original publications were contacted to confirm the VE estimates retained for analysis.

### Data Analysis

We abstracted the general design of the studies (case test-negative study, defined as a case-control study in which subjects with symptomatic influenza-like illness seeking medical care are tested by cultures or RT-PCR for influenza viruses [18]; other case-control study; or cohort study), the main specifications of the protocol (identification of the sponsor or institution that conducted the study, LAIV formulation, setting, eligibility criteria, and age range), the main specifications of the study operations (influenza season and country of enrollment), and the statistical analysis (adjustment for covariates or matching).

Unless otherwise specified, VE estimates retained for analysis were those for children aged 2–17 years, with the exclusion of children vaccinated <14 days before symptom onset and after adjustment to control for confounding at the study level. The point estimates and variances of the logarithm of the odds ratios, or relative risks for cohort studies, were derived from the effectiveness point estimates and 95% CIs of each study. Consolidated estimates of odds ratios across studies and then effectiveness estimates, defined as 100 × (1 – consolidated odds ratio or relative risk), were obtained with a random-effects model to take into account the risk of bias associated with the design of any of these observational studies. The *P* value of the test of heterogeneity between studies was presented when the consolidated VE estimate was significantly higher than 0.

Vaccine effectiveness estimates were categorized by LAIV formulation: monovalent, trivalent, and quadrivalent. One consolidated estimate was calculated for every season to determine effectiveness against any influenza strain, A(H1N1)pdm09 strains, A(H3N2) strains, and B strains. Multiseason consolidated estimates were also generated across all formulations. All statistical analyses were conducted using SAS version 9.3 (SAS Institute Inc., Cary, NC). We are not aware of any unpublished VE studies in outpatient children that could generate a publication bias.

## RESULTS

### Literature Search

**Figure 1.**
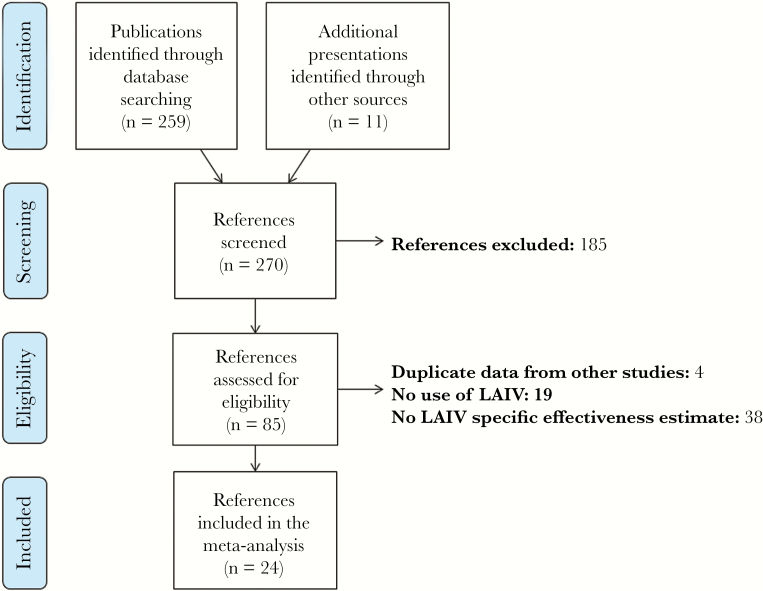
PRISMA flow diagram of systematic review. Abbreviation: LAIV, live-attenuated influenza vaccine.

We identified 259 unduplicated publications in peer-reviewed journals from the MEDLINE database (Figure 1). A further 11 potentially relevant presentations were found from searching the previously mentioned websites and reviewing reference lists. A total of 185 publications were excluded after screening because they did not present an original study or did not identify influenza cases by culture or RT-PCR; 85 publications met the eligibility criteria and were selected for full review (see Supplementary Material for list of eligible publications). A total of 24 references were retained for meta-analysis after the exclusion of 61 publications for the following reasons: duplicate data from other studies (n = 4), no use of LAIV (n = 19), and no LAIV-specific VE estimate (n = 38).

**Figure 2.**
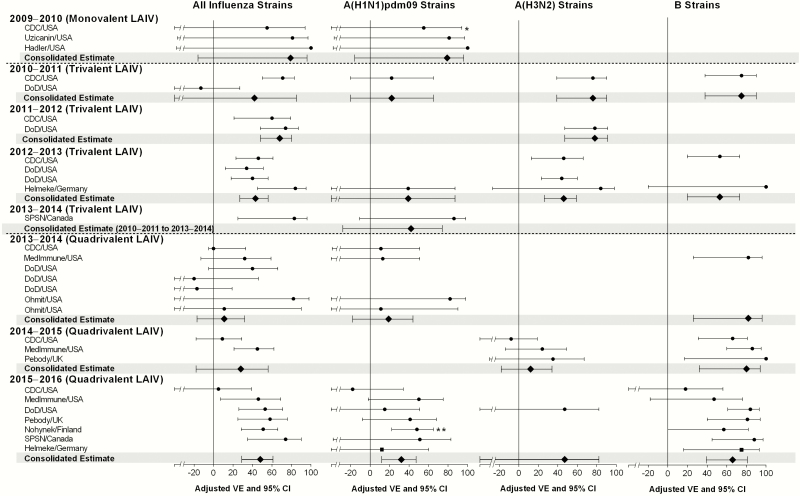
Vaccine effectiveness of live-attenuated influenza vaccine (LAIV)—estimates from original studies and consolidated estimates. *, LAIV vaccine effectiveness was 82% (95% confidence interval [CI], 14–96) if children were censored when they had received LAIV <7 days before nasal swab, instead of <14 days. **, Effectiveness against any A strains, with A(H1N1)pdm09 predominantly circulating. Abbreviations: CDC, Centers for Disease Control and Prevention; DoD, Department of Defense; SPSN, Sentinel Practitioner Surveillance Network; VE, vaccine effectiveness.

**Figure 3.**
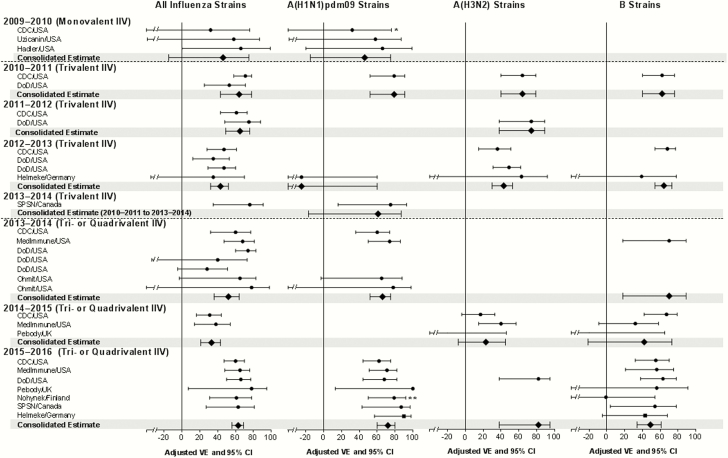
Vaccine effectiveness of inactivated influenza vaccine (IIV)—estimates from original studies and consolidated estimates. *, IIV vaccine effectiveness was 16% (95% confidence interval [CI], –108 to 66) if children were censored when they had received LAIV <7 days before nasal swab, instead of <14 days. **, Effectiveness against any A strains, with A(H1N1)pdm09 predominantly circulating. Abbreviations: CDC, Centers for Disease Control and Prevention; DoD, Department of Defense; SPSN, Sentinel Practitioner Surveillance Network; VE, vaccine effectiveness.

These 24 references presented a total of 29 different observational studies, with 2 references presenting 3 different studies and 1 reference presenting 2 studies. The characteristics of these studies are presented in Table 1. Of note, all but 4 studies were conducted according to a case test-negative design. The studies conducted by the US Department of Defense from 2010–2011 to 2013–2014 were conducted in active duty members and dependents. Because these studies did not report separate estimates for the pediatric and adult populations, the estimates assessed in the total population were consolidated into the analysis. Figures 2 and 3 as well as Supplementary Tables 1 and 2 present VE estimates against any influenza strain, A(H1N1)pdm09, A(H3N2), and B strains for LAIV and IIV, respectively.

### Effectiveness Against A(H1N1)pdm09 Strains

Using data in which cases occurring within 14 days of vaccination were excluded, 3 studies conducted in 2009–2010 suggested that the monovalent formulations of LAIV and IIV were effective against A(H1N1)pdm09 (79% [95% CI, –16 to 96] and 46% [95% CI, –15 to 75], respectively), but the sample sizes were too small for the VE estimates to be statistically significant. However, when cases occurring 8–14 days after vaccination were included in the CDC study, the VE of monovalent LAIV was effective with a consolidated estimate of 82% (95% CI, 14–96).

Trivalent LAIV was not shown to be effective in the United States during the 2010–2011 season (22%; 95% CI, < –20 to 65) or in Germany during the 2012–2013 season (39%; 95% CI, –176 to 87). Trivalent LAIV was shown to be effective in one study in 2013–2014. The consolidated estimate for LAIV3 formulations that contained the A/California/7/2009 vaccine strain from seasons 2010–2011 to 2013–2014 was 42% (95% CI, –28 to 74).

Similarly, LAIV4 was not shown to be effective in 2013–2014, with a consolidated estimate of 19% (95% CI, –18 to 44). However, it was effective in 2015–2016 (32%; 95% CI, 12–47) but significantly less effective than IIV (72%; 95% CI, 60–80). Consolidated estimates across seasons show that LAIV was effective as a quadrivalent formulation (29%; 95% CI, 14–42) and since the 2009 pandemic irrespective of the formulation (32%; 95% CI, 16–44).

### Effectiveness Against A(H3N2) Strains

Trivalent LAIV was effective in 2011–2012 (78%; 95% CI, 47–91), 2012–2013 (46%; 95% CI, 26–59), and in one study in 2010–2011 (76%; 95% CI, 39–90). As with IIV, LAIV4 was not effective in 2014–2015 when the circulating A(H3N2) strains were highly mismatched compared with the vaccine strains (12%; 95% CI, –18 to 34) [19]. Quadrivalent formulation of LAIV was also not effective in one study in 2015–2016 (47%; 95% CI, < –50 to 82); however, circulation of A(H3N2) strains was extremely limited. For the same reason, no effectiveness estimate was available in 2013–2014.

Consolidated estimates across seasons did not show LAIV to be effective as a quadrivalent formulation (15%; 95% CI, –13 to 36), but again the circulation of matched strains has remained extremely limited since 2013–2014. Live-attenuated influenza vaccine was effective as a trivalent formulation (59%; 95% CI, 39–73) and since the 2009 pandemic irrespective of the formulation (45%; 95% CI, 22–61).

### Effectiveness Against B Strains

Trivalent LAIV was effective against B strains in 2012–2013 (53%; 95% CI, 20–73) and in one study in 2010–2011 (75%; 95% CI, 38–90). No estimate was available for 2011–2012 because of low circulation of influenza B. The LAIV4 was effective in 2014–2015 (80%; 95% CI, 32–94), 2015–2016 (67%; 95% CI, 44–81), and in one study in 2013–2014 (82%; 95% CI, 26–96). Consolidated estimates across seasons show that LAIV was consistently effective as a trivalent formulation (62%; 95% CI, 32–79) and a quadrivalent formulation (75%; 95% CI, 57–85) and since the 2009 pandemic irrespective of the formulation (71%; 95% CI, 56–82).

### Effectiveness Against All Strains

Trivalent LAIV was significantly effective in 2011–2012 (68%; 95% CI, 48–80), 2012–2013 (43%; 95% CI, 27–56), and 2013–2014 (83%; 95% CI, 25–96). The LAIV4 was effective in 2015–2016 (48%; 95% CI, 29–61). The LAIV was not shown to be effective as a (1) monovalent formulation in 2009–2010 (79%; 95% CI, –16 to 96), (2) trivalent formulation in 2010–2011 (42%; 95% CI, –1 to 85), or (3) quadrivalent formulation in 2013–2014 (18%; 95% CI, –3 to 34) and 2014–2015 (28%; 95% CI, –18 to 56). Consolidated estimates across seasons show that LAIV was effective as a trivalent formulation (53%; 95% CI, 35–66) and a quadrivalent formulation (33%; 95% CI, 17–46) and since the 2009 pandemic irrespective of the formulation (42%; 95% CI, 30–52).

## DISCUSSION

The effectiveness of LAIV has not been consistently demonstrated since the 2009 pandemic. These findings are most clearly driven by suboptimal effectiveness against influenza A(H1N1)pdm09 strains. Live-attenuated influenza vaccine was not shown to be effective against A(H1N1)pdm09 strains in 2010–2011, 2012–2013, and 2013–2014, whether LAIV was distributed as a trivalent or quadrivalent formulation. The LAIV4 was effective against A(H1N1)pdm09 strains in 2015–2016 but with significantly reduced effectiveness relative to IIV. Effectiveness against influenza B strains was consistently observed. Trivalent LAIV was also effective against matched A(H3N2) strains, but there were insufficient data to estimate effectiveness against matched A(H3N2) of the quadrivalent formulation because of limited circulation of these strains in recent seasons. Similar to IIV, LAIV4 was not effective against mismatched A(H3N2) strains in 2014–2015.

The effectiveness data from recent seasons stand in contrast with the findings from randomized, double-blind clinical studies conducted in children aged 6 months to 7 years from influenza seasons 1996–1997 to 2002–2003 which showed that LAIV was efficacious versus placebo (pooled efficacy against all strains: 83% [95% CI, 69–91]) [20]. Moreover, compared with IIV, LAIV recipients aged 2–17 years enrolled in clinical studies experienced 44% (95% CI, 28–56) and 48% (95% CI, 38–57) fewer cases of influenza illness caused by vaccine-matched strains and all strains regardless of match to vaccine, respectively [21]. The superior efficacy of LAIV, compared with IIV, was observed for both antigenically well matched viruses and drifted A(H3N2) viruses.

The clear difference between the evidence from clinical studies before the 2009 pandemic and from observational studies after the 2009 pandemic raises questions about the validity of the findings from observational studies. However, the results of observational effectiveness studies before 2013 were generally consistent with the results of previous randomized studies, and the discrepant results since 2013–2014 may be completely explained by reduced effectiveness against A(H1N1)pdm09 strains. There are relatively large differences in LAIV effectiveness estimates between studies. In particular, there are consistent differences between effectiveness as estimated by the CDC Flu VE Network and the MedImmune-sponsored studies, both of which were conducted in the United States and had similar designs. Further research is needed to better understand these differences and whether they can be explained by variations between enrolled populations, local circulations of influenza strains, or other factors. Nevertheless, even given this heterogeneity, the data strongly suggest a suboptimal performance of LAIV against A(H1N1)pdm09 strains.

In addition to the studies reviewed here, which enrolled mostly individuals with outpatient illness, 2 more recent publications have reported LAIV effectiveness estimates against laboratory-confirmed influenza in a hospital setting in 2015–2016: VE in children aged 2–6 years in England was 54.5% (95% CI, 31.5–68.4) for all influenza types combined, 48.3% (95% CI, 16.9–67.8) for A(H1N1)pdm09, and 70.6% (95% CI, 33.2–87.1) for B strains [22]. Vaccine effectiveness against all laboratory-confirmed influenza in 4- to 11-year-olds in Scotland was 63% (95% CI, 50%–72%) [23].

Randomized studies have also been conducted since 2009 that provide information regarding the efficacy of LAIV. These studies include 3 prospective randomized controlled studies of the MedImmune LAIV since the 2009 pandemic: (1) a cluster-randomized, IIV-controlled study of school-aged children in Ontario, Canada and (2) a community-randomized, IIV-controlled study, both conducted in Hutterite communities in Canada, and (3) a placebo-controlled study in Japanese children. The first study was an open-label, cluster-randomized study involving 10 elementary schools, conducted in 2013–2014, that demonstrated greater protection with LAIV3 than trivalent IIV for both children and their household contacts compared with IIV during a season dominated by A(H1N1)pdm09 strains [24]. The second study was a larger cluster-randomized, blinded, IIV-controlled study of LAIV3 conducted between October 2012 and May 2015; the incidence rate of influenza cases among vaccine recipients did not differ by vaccine type [25]. The third study conducted in Japan enrolled more than 1200 Japanese children aged 7–18 years who were randomized to receive LAIV4 or placebo in 2014–2015, an influenza season with predominantly mismatched A(H3N2) strains. The LAIV4 efficacy against circulating A(H3N2) strains, all of which were mismatched to the vaccine strains, was 25.4% (95% CI, 4.3–41.7). Two randomized, placebo-controlled studies of a different LAIV3 construct using the Leningrad LAIV strains were also conducted in Bangladesh [26] and Senegal [27] in 2013, when the dominant circulating strains were A(H1N1)pdm09. The LAIV efficacy against all strains was 41% (95% CI, 28–52) in Bangladesh and –7% (95% CI, –34 to 15) in Senegal. Results from the randomized studies are generally consistent with those from the observational studies: efficacy against A(H1N1)pdm09 strains was not consistently demonstrated, and efficacy against mismatched A(H3N2) strains in 2014–2015 was significant but with a low point estimate. These results are similar to the findings of the MedImmune-sponsored effectiveness study in the United States and the United Kingdom effectiveness study in 2014–2015.

Environmental temperature exposure appears to have contributed to the suboptimal differences in LAIV effectiveness against A(H1N1)pdm09 strains in studies during 2013–2014 [28, 29], which is plausible given the known temperature sensitivity of the A/California/7/2009 wild-type and LAIV strains [30]. These findings led to the replacement of A/California/7/2009 (H1N1)pdm09 vaccine strain by the more thermostable A/Bolivia/559/2013 LAIV strain. Despite this change, LAIV effectiveness against A(H1N1)pdm09 remained low relative to IIV in 2015–2016.

Other hypotheses have also been raised to explain the recently observed suboptimal effectiveness of LAIV, particularly regarding the role of prior vaccination as well as potential interference between vaccine strains in the recently approved quadrivalent formulation. Available data do not support the hypothesis that vaccination in the previous season was associated with lower LAIV effectiveness. Point estimates of H1N1pdm09 VE among children vaccinated in the previous season trended higher in both 2013–2014 and 2015–2016 (Supplementary Table 3). In addition, negative effects of prior vaccination would be expected to effect influenza B effectiveness as well, which was not observed. Available data also do not support the hypothesis that specific interference associated with LAIV4 could explain the suboptimal effectiveness against A(H1N1)pdm09, because no significant effectiveness against A(H1N1)pdm09 was observed with LAIV3 from 2010–2011 to 2013–2014. As a result, a multifaceted research program has been initiated to systematically investigate the different phases of the LAIV virus life cycle, specifically viral entry, replication and assembly, budding, and spread. Preliminary findings have shown reduced replicative fitness of the A/California and A/Bolivia (H1N1)pdm09 LAIV strains compared with prepandemic A(H1N1) LAIV strains and other LAIV strains that previously demonstrated high efficacy in children (https://www.eventscribe.com/2017/NFIDACVR/ajaxcalls/PresentationInfo.asp?efp=SlVIV05JVUUzMjgw&PresentationID=253686&rnd=0.9800032).

Concern has been raised about the effectiveness of LAIV against A(H3N2) strains based on the results from the 2014–2015 season. The LAIV4 effectiveness in 2014–2015 against mismatched A(H3N2) strains, which were ≥8-fold different from the vaccine strain by hemagglutination inhibition assay (HAI), was low but similar to the effectiveness observed with IIV. Evidence of low but nonzero effectiveness of LAIV against these strains was substantiated by the randomized, placebo-controlled study of LAIV4 conducted in Japan in 2014–2015. The low LAIV4 VE against mismatched A(H3N2) strains in 2014–2015 was consistent with LAIV3 efficacy in previous randomized studies against mismatched A(H3N2) strains that were ≥8-fold different by HAI; VE has ranged from 18% to 31% (both estimates were nonsignificant) [4, 31]. This low efficacy against ≥8-fold mismatched A(H3N2) strains contrasted with the high efficacy of LAIV3 against mismatched A(H3N2) viruses that were 4- to 8-fold different by HAI [1]. Given the limited available data, it will be important to better understand the effectiveness of LAIV4 against matched A(H3N2) strains during the 2016–2017 influenza season, in which these are predominant, and in future seasons.

## CONCLUSIONS

This systematic review and meta-analysis demonstrates the reduced effectiveness of LAIV against influenza A(H1N1)pdm09 strains, which may explain the observations of inconsistent effectiveness of LAIV since the 2009 pandemic. This reduced A(H1N1)pdm09 effectiveness was observed with trivalent and quadrivalent formulations and whether children were previously vaccinated or not. Effectiveness against influenza B and matched A(H3N2) strains was consistently observed, although there were insufficient data to estimate effectiveness against matched A(H3N2) strains for the quadrivalent formulation because of limited circulation in recent seasons. A systematic investigation has been initiated to determine the root cause of the difference in effectiveness against pre- and postpandemic influenza A(H1N1) strains and to identify a more effective A(H1N1)pdm09 vaccine strain.

## Supplementary Data

Supplementary materials are available at *Open Forum Infectious Diseases* online. Consisting of data provided by the authors to benefit the reader, the posted materials are not copyedited and are the sole responsibility of the authors, so questions or comments should be addressed to the corresponding author.

## Supplementary Material

ofx111_suppl_Supplementary_Material
