# Supplementary material for: Live-Attenuated Influenza Vaccine Effectiveness in Children From 2009 to 2015–2016: A Systematic Review and Meta-Analysis
Source: Open Forum Infect Dis. 2017 Jul 24;4(3):ofx111. doi: 10.1093/ofid/ofx111 (PMC5569992; doi:10.1093/ofid/ofx111)
Supplement: ofx111_suppl_Supplementary_Material [file ofx111_suppl_supplementary_material.doc]

**SUPPLEMENTARY MATERIAL**

***Supplementary Table 1. Effectiveness of LAIV Against All Influenza Strains, A(H1N1)pdm09, A(H3N2), and B Strains and Number of LAIV Recipients Testing Positive for Influenza***

| **LAIV Formulations** | **Vaccine Effectiveness, % (95% CI)** | | | |
| --- | --- | --- | --- | --- |
|  | **All Strains** | **A(H1N1)pdm09 Strains** | **A(H3N2) Strains** | **B Strains** |
| ***Monovalent LAIV*** |  |  |  |  |
| 2009–2010 CDC/USA | 55 (–270 to 94) n = 1 | 55 (–270 to 94)^a^ n = 1 | — | — |
| 2009–2010 Uzicanin/USA | 81 (–37 to 97) n = 4 | 81 (–37 to 97) n = 4 | — | — |
| 2009–2010 Hadler/USA | 100 (–38 to 100) n = 0 | 100 (–38 to 100) n = 0 | — | — |
| **Consolidated estimate** | 79 (–16 to 96) | 79 (–16 to 96) | — | — |
| ***Trivalent LAIV*** |  |  |  |  |
| 2010–2011 CDC/USA | 71 (50–83) n = 22 | 22 (<–20 to 65)^c^ | 76 (39–90)^c^ | 75 (38–90)^c^ |
| 2010–2011 DoD/USA | –13 (–77 to 27)^c^ | — | — | — |
| **2010**–**2011 Consolidated estimate** | 42 (–119 to 85) | 22 (<–20 to 65) | 76 (39–90) | 75 (38–90) |
| 2011–2012 CDC/USA | 60 (21–79) n = 14 | — | — | — |
| 2011–2012 DoD/USA | 74 (48–87) n = 62 | — | 78 (47–91) n^c^ | — |
| **2011**–**2012 Consolidated estimate** | 68 (48–80)  *P* = 0.38^d^ | — | 78 (47–91) | — |
| 2012–2013 CDC/USA | 46 (23–61) n = 61 | — | 46 (13–66) n = 27 | 53 (20–73)^c^ |
| 2012–2013 DoD/USA (AFHSC) | 34 (12–51)^c^ | — | — | — |
| 2012–2013 DoD/USA (NHRC) | 40 (18–56)^c^ | — | — | — |
| 2012–2013 DoD/USA (USAFSAM) | — | — | 44 (23–60)^c^ | — |
| 2012–2013 Helmeke/Germany | 84 (45–95) n = 3 | 39 (–176 to 87)^c^ | 84 (–27 to 98)^c^ | 100 (–20 to 100)  n = 0 |
| **2012**–**2013 Consolidated estimate** | 43 (27–56)  *P* = 0.14^d^ | 39 (–176 to 87) | 46 (26–59)  *P* = 0.51^d^ | 53 (20–73) |
| 2013–2014 SPSN/Canada | 83 (25–96) n = 2 | 86 (–11 to 98) n = 1 | — | — |
| **Consolidated estimate, trivalent LAIV** | 53 (35–66) | 42 (–28 to 74) | 59 (39–73) | 62 (32–79) |
| ***Quadrivalent LAIV*** |  |  |  |  |
| 2013–2014 CDC/USA | –5 (<0–33)  n = 34 | 17 (–39 to 51)  n = 24 | — | — |
| 2013–2014 MedImmune/USA | 32 (–13 to 59) n = 30 | 13 (–55 to 51) n = 26 | — | 82 (26–96) n = 2 |
| 2013–2014 DoD/USA | 40 (–5 to 66)^c^ | — | — | — |
| 2013–2014 DoD/USA | –20 (–164 to 46)^c^ | — | — | — |
| 2013–2014 DoD/USA | –17 (–70 to 19)^c^ | — | — | — |
| 2013–2014 Ohmit/USA (2–8-year-olds) | 82 (–65 to 98) n = 1 | 82 (65 to 98) n = 1 | — | — |
| 2013–2014 Ohmit/USA (9–17-year-olds) | 11 (–658 to 90)  n = 1 | 11 (–658 to 90) n = 1 | — | — |
| **2013**–**2014 Consolidated estimate** | 11 (–17 to 32) | 19 (–18 to 44) | — | 82 (26–96) |
| 2014–2015 CDC/USA | 9 (–18 to 29)^c^ | — | –8 (–44 to 19) n = 102 | 66 (31–81)^c^ |
| 2014–2015 MedImmune | 45 (21–62) n = 51 | — | 24 (–14 to 49) n = 47 | 86 (60–95) n = 4 |
| 2014–2015 Public Health England | — | — | 35 (–30 to 67) n = 13 | 100 (17–100) n = 0 |
| **2014**–**2015 Consolidated estimate** | 28 (–18 to 56) | — | 12 (–18 to 34) | 80 (32–94)  *P* = 0.37^d^ |
| 2015–2016 CDC/USA | 5 (–48 to 39) n = 35 | –18 (<–50 to 34) n = 20 | — | 18 (<–50 to 56) n = 15 |
| 2015–2016 MedImmune/USA | 46 (7–69) n = 22 | 50 (–2 to 75) n = 12 | — | 47 (–18 to 76) n = 9 |
| 2015–2016 DoD/USA | 53 (26–71) n = 33 | 15 (–48 to 51) n = 23 | 47 (<–50 to 82) n = 4 | 84 (61–93) n = 6 |
| 2015–2016 Public Health England | 58 (25–76) n = 26 | 41 (–8 to 68) n = 22 | — | 81 (40–94) n = 4 |
| 2015–2016 Nohynek/Finland | 51 (28–66) n = 31 | 48 (22–65)^b^ n = 26 | — | 57 (0–82) n = 6 |
| 2015–2016 SPSN/Canada | 74 (35–90) n = 7 | 51 (–38 to 83) n = 5 | — | 88 (45–97) n = 2 |
| 2015–2016 Helmeke/Germany | — | 12 (–95 to 60) n = 9 | — | 75 (16–93)^c^ |
| **2015**–**2016 Consolidated estimate** | 48 (29–61)  *P* = 0.07^d^ | 32 (12–47)  *P* = 0.23^d^ | 47 (<–50 to 82) | 67 (44–81)  *P* = 0.02^d^ |
| **Consolidated estimate, quadrivalent LAIV** | 33 (17–46) | 29 (14–42) | 15 (–13 to 36) | 75 (57–85) |
| **Consolidated estimate, all LAIV formulations** | 42 (30–52) | 32 (16–44) | 45 (22–61) | 72 (57–82) |

Abbreviations: AFHSC, Armed Forces Health Surveillance Center; CDC, Centers for Disease Control and Prevention; CI, confidence interval; DoD, Department of Defense; LAIV, live attenuated influenza vaccine; NHRC, Naval Health Research Center; SPSN, Sentinel Practitioner Surveillance Network; USAFSAM, United States Air Force School of Aerospace Medicine.

^a^ LAIV vaccine effectiveness was 82% (95% CI, 14–96) if children were censored when they had received LAIV <7 days before nasal swab, instead of <14 days.

^b^ Effectiveness against any influenza A strains, with A(H1N1)pdm09 strains predominantly circulating; one child testing positive for A(H1N1)pdm09 and B strains.

^c^ Missing data.

^d^ *P* value of the heterogeneity test.

**Supplementary Table 2. Effectiveness of IIV Against All Influenza Strains, A(H1N1)pdm09, A(H3N2), and B Strains and Number of IIV Recipients Testing Positive for Influenza**

| **IIV Formulations** | **Vaccine Effectiveness, % (95% CI)** | | | |
| --- | --- | --- | --- | --- |
|  | **All Strains** | **A(H1N1)pdm09 Strains** | **A(H3N2) Strains** | **B Strains** |
| ***Monovalent IIV*** |  |  |  |  |
| 2009–2010 CDC/USA | 32 (–92 to 76) n = 5 | 32 (–92 to 76)^a^ n = 5 | — | — |
| 2009–2010 Uzicanin/USA | 58 (–39 to 87) n = 19 | 58 (–39 to 87) n = 19 | — | — |
| 2009–2010 Hadler/USA | 66 (–116 to 99) n = 1 | 66 (–116 to 99) n = 1 | — | — |
| **2009–2010 Consolidated estimate** | 46 (–15 to 75) | 46 (–15 to 75) | — | — |
| ***Trivalent IIV*** |  |  |  |  |
| 2010–2011 CDC/USA | 71 (58–78) n = 66 | 79 (52–91)^c^ | 64 (40–79)^c^ | 62 (40–76)^c^ |
| 2010–2011 DoD/USA | 53 (25–71)^c^ | — | — | — |
| **2010**–**2011 Consolidated estimate** | 64 (43–78)  *P* = 0.09^d^ | 79 (52–91) | 64 (40–79) | 62 (40–76) |
| 2011–2012 CDC/USA | 61 (43–73) n = 58 | — | — | — |
| 2011–2012 DoD/USA | 75 (48–88) n = 29 | — | 74 (38–89) n = 29 | — |
| **2011**–**2012 Consolidated estimate** | 65 (49–76)  *P* = 0.29^d^ | — | 74 (38–89) | — |
| 2012–2013 CDC/USA | 47 (28–61) n = 198 | — | 36 (15–51) n = 99 | 68 (54–77) ^c^ |
| 2012–2013 DoD/USA (AFHSC) | 35 (12–53)^c^ | — | — | — |
| 2012–2013 DoD/USA (NHRC) | 47 (29–60)^c^ | — | — | — |
| 2012–2013 DoD/USA (USAFSAM) | — | — | 49 (31–62)^c^ | — |
| 2012–2013 Helmeke/Germany | 35 (–35 to 70) n = 12 | –25 (–296 to 60)^c^ | 63 (–67 to 92)^c^ | 39 (–66 to 78)^c^ |
| **2012**–**2013 Consolidated estimate** | 43 (32–52)  *P* = 0.74^d^ | –25 (–296 to 60) | 43 (30–53)  *P* = 0.46^d^ | 64 (54–73)  *P* = 0.24^d^ |
| 2013–2014 SPSN/Canada | 76 (35–91) n = 5 | 75 (16–93) n = 3 | — | — |
| **Consolidated estimate from 2010**–**2011 to 2013**–**2014** | 56 (44–65) | 61 (–17 to 87) | 52 (36–63) | 64 (54–78) |
| ***Trivalent or quadrivalent IIV*** |  |  |  |  |
| 2013–2014 CDC/USA | 60 (32–77) n = 37 | 60 (36–74) n = 27 | — | — |
| 2013–2014 MedImmune/USA | 68 (47–81) n = 24 | 74 (50–86) n = 14 | — | 70 (18–89) n = 6 |
| 2013–2014 DoD/USA | 74 (60–83)^c^ | — | — | — |
| 2013–2014 DoD/USA | 40 (–34 to 73)^c^ | — | — | — |
| 2013–2014 DoD/USA | 28 (–5 to 51)^c^ | — | — | — |
| 2013–2014 Ohmit/USA (2–8-year-olds) | 65 (–3 to 83) n = 6 | 65 (–3 to 88) n = 6 | — | — |
| 2013–2014 Ohmit/USA (9–17-year-olds) | 78 (–150 to 98) n = 1 | 78 (–150 to 98) n = 1 | — | — |
| **2013**–**2014 Consolidated estimate** | 52 (36–64)  *P* = 0.02^d^ | 66 (52–75)  *P* = 0.73^d^ | — | 70 (18–89) |
| 2014–2015 CDC/USA | 31 (16–44) n^c^ | — | 17 (–4 to 33) n = 165 | 67 (42–79) n = 17 |
| 2014–2015 MedImmune | 38 (14–54) n = 105 | — | 40 (15–57) n = 71 | 32 (–9 to 58) n = 34 |
| 2014–2015 Public Health England | — | — | –73 (–457 to 46) n = 6 | –124 (–1343 to 65) n = 2 |
| **2014**–**2015 Consolidated estimate** | 33 (21–43)  *P* = 0.57^d^ | — | 23 (–8 to 45) | 42 (–21 to 73) |
| 2015–2016 CDC/USA | 60 (47–70) n = 73 | 62 (44–75) n = 38 | — | 55 (32–70) n = 35 |
| 2015–2016 MedImmune/USA | 65 (48–76) n = 44 | 71 (51–82) n = 25 | — | 56 (21–75) n = 19 |
| 2015–2016 DoD/USA | 66 (50–77) n = 42 | 68 (44–82) n = 18 | 82 (38–95) n = 3 | 63 (38–78) n = 21 |
| 2015–2016 Public Health England | 78 (7–95) n = 3 | 100 (13–100) n = 0 | — | 56 (–122 to 91) n = 3 |
| 2015–2016 Nohynek/Finland | 61 (31–78) n = 12 | 79 (50–92)^b^ n = 5 | — | –1 (–123 to 54) n = 7 |
| 2015–2016 SPSN/Canada | 63 (27–81) n = 15 | 87 (43–97) n = 4 | — | 54 (4–78) n = 11 |
| 2015–2016 Helmeke/Germany | — | 90 (57–98) n = 2 | — | 43 (–5 to 68) n=^c^ |
| **2015**–**2016 Consolidated estimate** | 63 (56–69)  *P* = 0.95^d^ | 72 (60–80)  *P* = 0.45^d^ | 82 (38–95) | 49 (34–61)  *P* = 0.67^d^ |
| **Consolidated estimate from 2013**–**2014 to 2015**–**2016** | 57 (47–65) | 68 (60–74) | 33 (–11 to 60) | 50 (47–60) |
| **Consolidated estimate, all IIV formulations** | 56 (49–62) | 67 (60–73) | 46 (28–59) | 55 (45–63) |

Abbreviations: AFHSC, Armed Forces Health Surveillance Center; CDC, Centers for Disease Control and Prevention; CI, confidence interval; DoD, Department of Defense; IIV, inactivated influenza vaccine; NHRC, Naval Health Research Center; SPSN, Sentinel Practitioner Surveillance Network; USAFSAM, United States Air Force School of Aerospace Medicine.

^a^ IIV vaccine effectiveness was 16% (95% CI, –108 to 66) if children were censored when they had received LAIV <7 days before nasal swab, instead of <14 days.

^b^ Effectiveness against any A strains, with A(H1N1)pdm09 predominantly circulating.

^c^ Missing data.

^d^ *P* value of the heterogeneity test.

**Supplementary Table 3. Effectiveness of LAIV and IIV Against All Influenza Strains as a Function of Vaccination in the Previous Season**

|  | **Adjusted LAIV Effectiveness  % (95% CI)** | **Adjusted IIV Effectiveness  % (95% CI)** |
| --- | --- | --- |
|
|
| **MedImmune 2013–2014** | | |
| Not vaccinated against influenza in 2012–2013 | 9 (–161 to 68) | 83 (39–95) |
| Vaccinated against influenza in 2012–2013 | 19 (–80 to 64) | 64 (15–85) |
| **MedImmune 2015–2016** |  |  |
|
|
| Not vaccinated against influenza in 2014–2015 | 35 (–206 to 86) | 49 (–33 to 81) |
| Vaccinated against influenza in 2014–2015 | 60 (1–84) | 79 (57–90) |
| **Nohynek/Finland 2015–2016** |  |  |
| Not vaccinated against influenza in 2014–2015 | 25 (–27 to 56) | 71 (–18 to 93) |
| Vaccinated against influenza in 2014–2015 | 74 (48–87) | 87 (57–96) |
| Consolidated estimates |  |  |
| Children not vaccinated in previous season | 23 (–21 to 51) | 67 (36–83) |
| Children vaccinated in previous season | 57 (15–78) | 77 (62–86) |

Abbreviations: CI, confidence interval; IIV, inactivated influenza vaccine; LAIV, live attenuated influenza vaccine.

**Supplementary Material: List of Eligible Publications Identified During the Literature Search**

**Influenza Season 2009–2010**

1. Griffin MR, Monto AS, Belongia EA, et al. Effectiveness of non-adjuvanted pandemic influenza A vaccines for preventing pandemic influenza acute respiratory illness visits in 4 U.S. communities. PLoS One **2011**; 6:e23085.
2. Uzicanin A, Thompson M, Smith P, et al. Effectiveness of 1 dose of influenza A (H1N1) 2009 monovalent vaccines in preventing reverse-transcription polymerase chain reaction-confirmed H1N1 infection among school-aged children in Maine. J Infect Dis **2012**; 206:1059–68.
3. Hadler JL, Baker TN, Papadouka V, et al. Effectiveness of 1 dose of 2009 influenza A (H1N1) vaccine at preventing hospitalization with pandemic H1N1 influenza in children aged 7 months-9 years. J Infect Dis **2012**; 206:49–55.

**Influenza Season 2010–2011**

1. Treanor JJ, Talbot HK, Ohmit SE, et al. Effectiveness of seasonal influenza vaccines in the United States during a season with circulation of all three vaccine strains. Clin Infect Dis **2012**; 55:951–9.
2. Eick-Cost AA, Tastad KJ, Guerrero AC, et al. Effectiveness of seasonal influenza vaccines against influenza-associated illnesses among US military personnel in 2010-11: a case-control approach. PLoS One **2012**; 7:e41435.

**Influenza Season 2011–2012**

1. Ohmit SE, Thompson MG, Petrie JG, et al. Influenza vaccine effectiveness in the 2011-2012 season: protection against each circulating virus and the effect of prior vaccination on estimates. Clin Infect Dis **2014**; 58:319–27.
2. MacIntosh VH, Tastad KJ, Eick-Cost AA. Mid-season influenza vaccine effectiveness 2011-2012: a Department of Defense global, laboratory-based, influenza surveillance system case-control study estimate. Vaccine **2013**; 31:1651–5.

**Influenza Season 2012–2013**

1. McLean HQ, Thompson MG, Sundaram ME, et al. Influenza vaccine effectiveness in the United States during 2012-2013: variable protection by age and virus type. J Infect Dis **2015**; 211:1529–40.
2. Eick-Cost AA, Hu Z, Cooper MJ, et al. Mid-season influenza vaccine effectiveness for the 2012-2013 influenza season. MSMR **2013**; 20:15–6.
3. Helmeke C, Grafe L, Irmscher HM, Gottschalk C, Karagiannis I, Oppermann H. Effectiveness of the 2012/13 trivalent live and inactivated influenza vaccines in children and adolescents in Saxony-Anhalt, Germany: a test-negative case-control study. PLoS One **2015**; 10:e0122910.

**Influenza Season 2013–2014**

1. Gaglani M, Pruszynski J, Murthy K, et al. Influenza vaccine effectiveness against 2009 pandemic influenza A(H1N1) virus differed by vaccine type during 2013-2014 in the United States. J Infect Dis **2016**; 213:1546–56.
2. Caspard H, Gaglani M, Clipper L, et al. Effectiveness of live attenuated influenza vaccine and inactivated influenza vaccine in children 2-17 years of age in 2013-2014 in the United States. Vaccine **2016**; 34:77–82.
3. Skowronski DM, Chambers C, Sabaiduc S, et al. Integrated sentinel surveillance linking genetic, antigenic, and epidemiologic monitoring of influenza vaccine-virus relatedness and effectiveness during the 2013-2014 influenza season. J Infect Dis **2015**; 212:726–39.
4. Cost AA, Hiser MJ, Hu Z, et al. Brief report: mid-season influenza vaccine effectiveness estimates for the 2013-2014 influenza season. MSMR **2014**; 21:15–7.
5. Ohmit SE, Petrie JG, Malosh RE, et al. Substantial influenza vaccine effectiveness in households with children during the 2013-2014 influenza season, when 2009 pandemic influenza A(H1N1) virus predominated. J Infect Dis **2016**; 213:1229–36.

**Influenza Season 2014–2015**

1. Clippard J, Flannery B. End-of-season influenza vaccine effectiveness estimates for the 2014-15 season: US Influenza Vaccine Effectiveness (Flu VE) Network. Presented at the Advisory Committee on Immunization Practices; June 24, 2015; Atlanta, GA. Available at: <http://www.cdc.gov/vaccines/acip/meetings/downloads/slides-2015-06/flu-02-flannery.pdf>. Accessed 16 February 2017.
2. Caspard H, Gaglani M, Clipper L, et al. Multicenter study of live-attenuated influenza vaccine effectiveness in children, 2014–15. Open Forum Infect Dis **2015**; 2:1352.
3. Pebody R, Warburton F, Andrews N, et al. Effectiveness of seasonal influenza vaccine in preventing laboratory-confirmed influenza in primary care in the United Kingdom: 2014/15 end of season results. Euro Surveill **2015**; 20.

**Influenza Season 2015–2016**

1. Flannery B, Chung J. Influenza vaccine effectiveness, including LAIV vs IIV in children and adolescents, US Flu VE Network, 2015-16. Presented at the Advisory Committee on Immunization Practices; June 22, 2016; Atlanta, GA. Available at: <https://www.cdc.gov/vaccines/acip/meetings/downloads/slides-2016-06/influenza-05-flannery.pdf>. Accessed 16 February 2017.
2. Ambrose C. 2015-16 US Influenza Vaccine Effectiveness Influenza Clinical Investigation for Children (ICICLE) Study. Presented at the Advisory Committee on Immunization Practices; June 22, 2016; Atlanta, GA. Available at: <https://www.cdc.gov/vaccines/acip/meetings/downloads/slides-2016-06/influenza-06-ambrose.pdf>. Accessed 16 February 2017.
3. Nohynek H, Baum U, Syrjanen R, Ikonen N, Sundman J, Jokinen J. Effectiveness of the live attenuated and the inactivated influenza vaccine in two-year-olds - a nationwide cohort study Finland, influenza season 2015/16. Euro Surveill **2016**; 21.
4. Pebody R, Warburton F, Ellis J, et al. Effectiveness of seasonal influenza vaccine for adults and children in preventing laboratory-confirmed influenza in primary care in the United Kingdom: 2015/16 end-of-season results. Euro Surveill **2016**; 21.
5. Skworonski D. Live attenuated influenza vaccine (LAIV) vs. inactivated influenza vaccine (IIV): summary of effectiveness evidence since 2009. Presented to the National Advisory Committee on Immunization Influenza Working Group; February 10–11, 2016; Ottawa, Ontario. Available at: <http://www.phac-aspc.gc.ca/naci-ccni/flu-2016-grippe-addendum-children-enfants-eng.php>. Accessed 16 February 2017.
6. Helmeke C. Effektivität der Influenza-Impfstoffe bei Kindern in Sachsen-Anhalt und Niedersachsen 2015/16. Available at: <http://www.verbraucherschutz.sachsen-anhalt.de/fileadmin/Bibliothek/Politik_und_Verwaltung/MS/LAV_Verbraucherschutz/hygiene/influenza/Effektivitaet_der_Influenzaimpfstoffe_2015-16.pdf>. Accessed 16 February 2017.

**Duplicate Data From Other Publications/Presentations**

1. Zimmerman RK, Nowalk MP, Chung J, et al. 2014-2015 Influenza vaccine effectiveness in the United States by vaccine type. Clin Infect Dis **2016**; 63:1564–73.
2. Chung JR, Flannery B, Thompson MG, et al. Seasonal effectiveness of live attenuated and inactivated influenza vaccine. Pediatrics **2016**; 137:e20153279.
3. Hermann N. [Effectiveness of live attenuated influenza vaccines and trivalent inactivated influenza vaccines against confirmed influenza in children and adolescents in Saxony-Anhalt, 2012/13)]. Gesundheitswesen **2015**; 77:499–501.
4. Flannery B. Comparative effectiveness of LAIV and TIV in 2013-14 in the US Flu VE Network. Presented at the Meeting to Assess Live-Attenuated Influenza Vaccines to Prevent Pediatric Influenza Disease in Low and Middle Income Countries; December 17, 2014; Geneva.

**No Use of Live Attenuated Influenza Vaccine**

1. Andrews N, McMenamin J, Durnall H, et al. Effectiveness of trivalent seasonal influenza vaccine in preventing laboratory-confirmed influenza in primary care in the United Kingdom: 2012/13 end of season results. Euro Surveill **2014**; 19:5–13.
2. Castilla J, Martinez-Artola V, Salcedo E, et al. Vaccine effectiveness in preventing influenza hospitalizations in Navarre, Spain, 2010-2011: cohort and case-control study. Vaccine **2012**; 30:195–200.
3. Castilla J, Martinez-Baz I, Martinez-Artola V, et al. Early estimates of influenza vaccine effectiveness in Navarre, Spain: 2012/13 mid-season analysis. Euro Surveill **2013**; 18:2.
4. Castilla J, Martinez-Baz I, Martinez-Artola V, et al. Decline in influenza vaccine effectiveness with time after vaccination, Navarre, Spain, season 2011/12. Euro Surveill **2013**; 18.
5. Castilla J, Martinez-Baz I, Navascues A, et al. Vaccine effectiveness in preventing laboratory-confirmed influenza in Navarre, Spain: 2013/14 mid-season analysis. Euro Surveill **2014**; 19.
6. Gefenaite G, Rahamat-Langendoen J, Ambrozaitis A, et al. Seasonal influenza vaccine effectiveness against influenza in 2012-2013: a hospital-based case-control study in Lithuania. Vaccine **2014**; 32:857–63.
7. Kim KH, Choi YG, Yoon HB, et al. Evaluation of the effectiveness of pandemic influenza A(H1N1) 2009 vaccine based on an outbreak investigation during the 2010-2011 season in Korean military camps. Osong Public Health Res Perspect **2013**; 4:209–14.
8. Kissling E, Valenciano M, Buchholz U, et al. Influenza vaccine effectiveness estimates in Europe in a season with three influenza type/subtypes circulating: the I-MOVE multicentre case-control study, influenza season 2012/13. Euro Surveill **2014**; 19.
9. Leval A, Hergens MP, Persson K, Ortqvist A. Real-time real-world analysis of seasonal influenza vaccine effectiveness: method development and assessment of a population-based cohort in Stockholm County, Sweden, seasons 2011/12 to 2014/15. Euro Surveill **2016**; 21.
10. Ohmit SE, Petrie JG, Malosh RE, et al. Influenza vaccine effectiveness in the community and the household. Clin Infect Dis **2013**; 56:1363–9.
11. Simpson CR, Lone N, Kavanagh K, et al. Seasonal Influenza Vaccine Effectiveness (SIVE): an observational retrospective cohort study - exploitation of a unique community-based national-linked database to determine the effectiveness of the seasonal trivalent influenza vaccine. Southampton UK: NIHR Journals Library; 2013.
12. Skowronski DM, Janjua NZ, De Serres G, et al. A sentinel platform to evaluate influenza vaccine effectiveness and new variant circulation, Canada 2010-2011 season. Clin Infect Dis **2012**; 55:332–42.
13. Song JY, Cheong HJ, Heo JY, et al. Effectiveness of the pandemic influenza A/H1N1 2009 monovalent vaccine in Korea. Vaccine **2011**; 29:1395–8.
14. Costanzo S, Gianfagna F, Persichillo M, et al. Pandemic and seasonal vaccine coverage and effectiveness during the 2009-2010 pandemic influenza in an Italian adult population. Int J Public Health **2012**; 57:569–79.
15. Martinez-Baz I, Guevara M, Elia F, Ezpeleta C, Fernandez Alonso M, Castilla J. [Influence of distinct criteria for selecting patients for swabbing on estimation of the effectiveness of the influenza vaccine). Gac Sanit **2014**; 28:225–9.
16. Fu C, Xu J, Lin J, et al. Concurrent and cross-season protection of inactivated influenza vaccine against A(H1N1)pdm09 illness among young children: 2012-2013 case-control evaluation of influenza vaccine effectiveness. Vaccine **2015**; 33:2917–21.
17. Nakata K, Fujieda M, Miki H, et al. Detection of influenza vaccine effectiveness among nursery school children: lesson from a season with cocirculating respiratory syncytial virus. Hum Vaccin Immunother **2015**; 11:545–52.
18. McLean HQ, Thompson MG, Sundaram ME, et al. Impact of repeated vaccination on vaccine effectiveness against influenza A(H3N2) and B during 8 seasons. Clin Infect Dis **2014**; 59:1375–85.
19. McMenamin J, Andrews N, Robertson C, et al. Effectiveness of seasonal 2012/13 vaccine in preventing laboratory-confirmed influenza infection in primary care in the United Kingdom: mid-season analysis 2012/13. Euro Surveill **2013**; 18.

**No LAIV-Specific Effectiveness Estimate**

1. Skowronski DM, Janjua NZ, De Serres G, et al. Interim estimates of influenza vaccine effectiveness in 2012/13 from Canada's sentinel surveillance network, January 2013. Euro Surveill **2013**; 18.
2. Skowronski DM, Janjua NZ, De Serres G, et al. Low 2012-13 influenza vaccine effectiveness associated with mutation in the egg-adapted H3N2 vaccine strain not antigenic drift in circulating viruses. PLoS One **2014**; 9:e92153.
3. Skowronski DM, Janjua NZ, Sabaiduc S, et al. Influenza A/subtype and B/lineage effectiveness estimates for the 2011-2012 trivalent vaccine: cross-season and cross-lineage protection with unchanged vaccine. J Infect Dis **2014**; 210:126–37.
4. Ohmit SE, Petrie JG, Malosh RE, Fry AM, Thompson MG, Monto AS. Influenza vaccine effectiveness in households with children during the 2012-2013 season: assessments of prior vaccination and serologic susceptibility. J Infect Dis **2015**; 211:1519–28.
5. Chambers C, Skowronski DM, Sabaiduc S, et al. Interim estimates of 2015/16 vaccine effectiveness against influenza A(H1N1)pdm09, Canada, February 2016. Euro Surveill **2016**; 21:30168.
6. Ferdinands JM, Olsho LE, Agan AA, et al. Effectiveness of influenza vaccine against life-threatening RT-PCR-confirmed influenza illness in US children, 2010-2012. J Infect Dis **2014**; 210:674–83.
7. Flannery B, Clippard J, Zimmerman RK, et al. Early estimates of seasonal influenza vaccine effectiveness - United States, January 2015. MMWR Morb Mortal Wkly Rep **2015**; 64:10–5.
8. Flannery B, Zimmerman RK, Gubareva LV, et al. Enhanced genetic characterization of influenza A(H3N2) viruses and vaccine effectiveness by genetic group, 2014-2015. J Infect Dis **2016**; 214:1010–9.
9. Jimenez-Jorge S, Pozo F, de Mateo S, et al. Influenza vaccine effectiveness in Spain 2013/14: subtype-specific early estimates using the cycEVA study. Euro Surveill **2014**; 19.
10. Kissling E, Nunes B, Robertson C, et al. I-MOVE multicentre case-control study 2010/11 to 2014/15: Is there within-season waning of influenza type/subtype vaccine effectiveness with increasing time since vaccination? Euro Surveill **2016**; 21.
11. Menniti-Ippolito F, Da Cas R, Traversa G, et al. Vaccine effectiveness against severe laboratory-confirmed influenza in children: results of two consecutive seasons in Italy. Vaccine **2014**; 32:4466–70.
12. Cowling BJ, Feng S, Finelli L, Steffens A, Fowlkes A. Assessment of influenza vaccine effectiveness in a sentinel surveillance network 2010-13, United States. Vaccine **2016**; 34:61–6.
13. Skowronski DM, Chambers C, Sabaiduc S, et al. A perfect storm: impact of genomic variation and serial vaccination on low influenza vaccine effectiveness during the 2014-2015 season. Clin Infect Dis **2016**; 63:21–32.
14. Skowronski D, Chambers C, Sabaiduc S, et al. Interim estimates of 2013/14 vaccine effectiveness against influenza A(H1N1)pdm09 from Canada’s sentinel surveillance network, January 2014. Euro Surveill **2014**; 19.
15. Smithgall M, Vargas CY, Reed C, et al. Influenza vaccine effectiveness in a low-income, urban community cohort. Clin Infect Dis **2016**; 62:358–60.
16. Radin JM, Hawksworth AW, Myers CA, Ricketts MN, Hansen EA, Brice GT. Influenza vaccine effectiveness: maintained protection throughout the duration of influenza seasons 2010-2011 through 2013-2014. Vaccine **2016**; 34:3907–12.
17. Valenciano M, Kissling E, Reuss A, Jimenez-Jorge S, Horvath JK, Donnell JM, et al. The European I-MOVE Multicentre 2013-2014 Case-Control Study. Homogeneous moderate influenza vaccine effectiveness against A(H1N1)pdm09 and heterogenous results by country against A(H3N2). Vaccine **2015**;33(24):2813-22.
18. Valenciano M, Kissling E, Reuss A, Rizzo C, Gherasim A, Horvath JK, et al. Vaccine effectiveness in preventing laboratory-confirmed influenza in primary care patients in a season of co-circulation of influenza A(H1N1)pdm09, B and drifted A(H3N2), I-MOVE Multicentre Case-Control Study, Europe 2014/15. Euro Surveill **2016**;21(7):pii=30139
19. Lytras T, Kossyvakis A, Melidou A, Andreopoulou A, Exindari M, Gioula G, et al. Influenza vaccine effectiveness in preventing hospitalizations with laboratory-confirmed influenza in Greece during the 2014-2015 season: A test-negative study. J Med Virol **2016**;88(11):1896-904.
20. Lytras T, Kossyvakis A, Melidou A, Exindari M, Gioula G, Pogka V, et al. Influenza vaccine effectiveness against laboratory confirmed influenza in Greece during the 2013-2014 season: a test-negative study. Vaccine **2015**;33(2):367-73
21. Martinez-Baz I, Navascues A, Pozo F, Chamorro J, Albeniz E, Casado I, et al. Influenza vaccine effectiveness in preventing inpatient and outpatient cases in a season dominated by vaccine-matched influenza B virus. Hum Vaccin Immunother **2015**;11(7):1626-33.
22. Souty C, Blanchon T, Bonmarin I, Levy-Bruhl D, Behillil S, Enouf V, et al. Early estimates of 2014/15 seasonal influenza vaccine effectiveness in preventing influenza-like illness in general practice using the screening method in France. Hum Vaccin Immunother **2015**;11(7):1621-5
23. Nunes B, Machado A, Guiomar R, Pechirra P, Conde P, Cristovao P, et al. Estimates of 2012/13 influenza vaccine effectiveness using the case test-negative control design with different influenza negative control groups. Vaccine **2014**;32(35):4443-9.
24. Puig-Barbera J, Natividad-Sancho A, Launay O, Burtseva E, Ciblak MA, Tormos A, et al. 2012-2013 Seasonal influenza vaccine effectiveness against influenza hospitalizations: results from the global influenza hospital surveillance network. PLoS One **2014**;9(6):e100497.
25. Rizzo C, Bella A, Alfonsi V, Puzelli S, Palmieri AP, Chironna M, et al. Influenza vaccine effectiveness in Italy: Age, subtype-specific and vaccine type estimates 2014/15 season. Vaccine **2016**;34(27):3102-8.
26. Song JY, Lee JS, Wie SH, Kim HY, Lee J, Seo YB, et al. Prospective cohort study on the effectiveness of influenza and pneumococcal vaccines in preventing pneumonia development and hospitalization. Clin Vaccine Immunol **2015**;22:229–34.
27. Torner N, Martinez A, Basile L, et al. Influenza vaccine effectiveness assessment through sentinel virological data in three post-pandemic seasons. Hum Vaccin Immunother **2015**; 11:225–30.
28. Valenciano M, Kissling E. Early estimates of seasonal influenza vaccine effectiveness in Europe: results from the I-MOVE multicentre case-control study, 2012/13. Euro Surveill **2013**; 18:3.
29. Zimmerman RK, Nowalk MP, Lin CJ, et al. Cluster randomized trial of a toolkit and early vaccine delivery to improve childhood influenza vaccination rates in primary care. Vaccine **2014**; 32:3656–63.
30. Thompson MG, Clippard J, Petrie JG, et al. Influenza vaccine effectiveness for fully and partially vaccinated children 6 months to 8 years old during 2011-2012 and 2012-2013: the importance of two priming doses. Pediatr Infect Dis J **2016**; 35:299–308.
31. Thompson MG, Sokolow LZ, Almendares O, et al. Effectiveness of nonadjuvanted monovalent influenza A(H1N1)pdm09 vaccines for preventing reverse transcription polymerase chain reaction-confirmed pandemic influenza hospitalizations: case-control study of children and adults at 10 US influenza surveillance network sites. Clin Infect Dis **2013**; 57:1587–92.
32. Bonmarin I, Belchior E, Le Strat Y, Levy-Bruhl D. First estimates of influenza vaccine effectiveness among severe influenza cases, France, 2011/12. Euro Surveill **2012**; 17.
33. Pebody R, Warburton F, Andrews N, et al. Effectiveness of seasonal influenza vaccine in preventing laboratory-confirmed influenza in primary care in the United Kingdom: 2014/15 end of season results. Euro Surveill **2015**; 20.
34. Ojha RP, Stallings-Smith S, Flynn PM, Adderson EE, Offutt-Powell TN, Gaur AH. The impact of vaccine concerns on racial/ethnic disparities in influenza vaccine uptake among health care workers. Am J Public Health **2015**; 105:e35–41.
35. Woolpert T, Phillips CJ, Sevick C, Crum-Cianflone NF, Blair PJ, Faix D. Health-related behaviors and effectiveness of trivalent inactivated versus live attenuated influenza vaccine in preventing influenza-like illness among young adults. PLoS One **2014**; 9:e102154.
36. Phillips CJ, Woolpert T, Sevick C, Faix D, Blair PJ, Crum-Cianflone NF. Comparison of the effectiveness of trivalent inactivated influenza vaccine and live, attenuated influenza vaccine in preventing influenza-like illness among US military service members, 2006-2009. Clin Infect Dis **2013**; 56:11–9.
37. Johns MC, Eick AA, Blazes DL, et al. Seasonal influenza vaccine and protection against pandemic (H1N1) 2009-associated illness among US military personnel. PLoS One **2010**; 5:e10722.
38. Bateman AC, Kieke BA, Irving SA, Meece JK, Shay DK, Belongia EA. Effectiveness of monovalent 2009 pandemic influenza A virus subtype H1N1 and 2010-2011 trivalent inactivated influenza vaccines in Wisconsin during the 2010-2011 influenza season. J Infect Dis **2013**; 207:1262–9.
